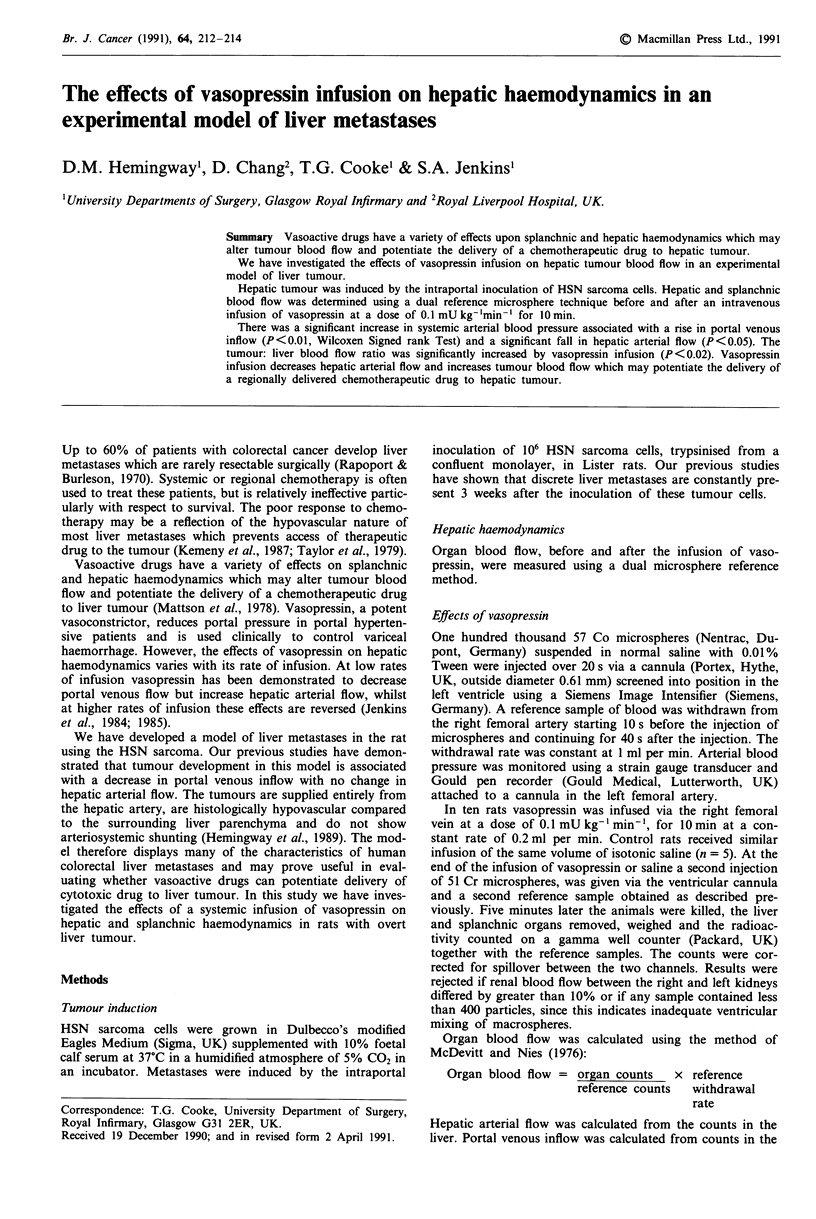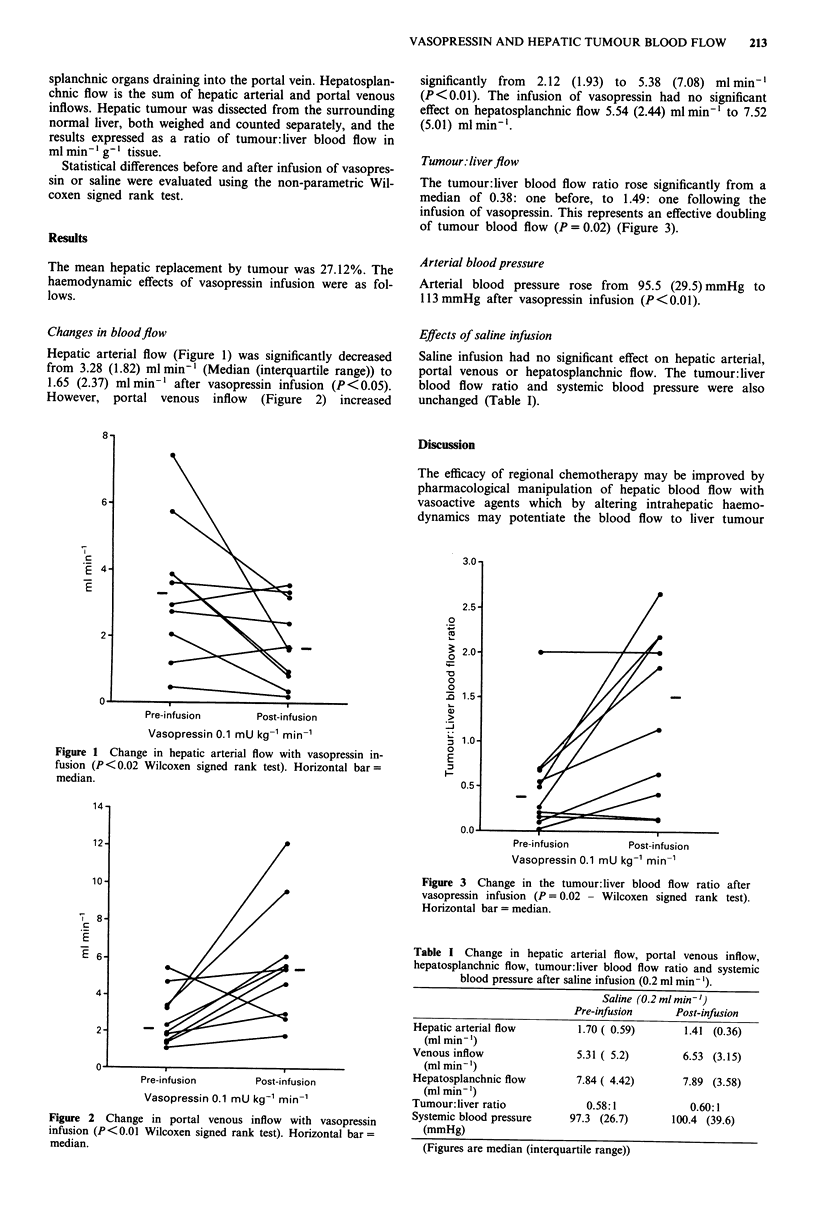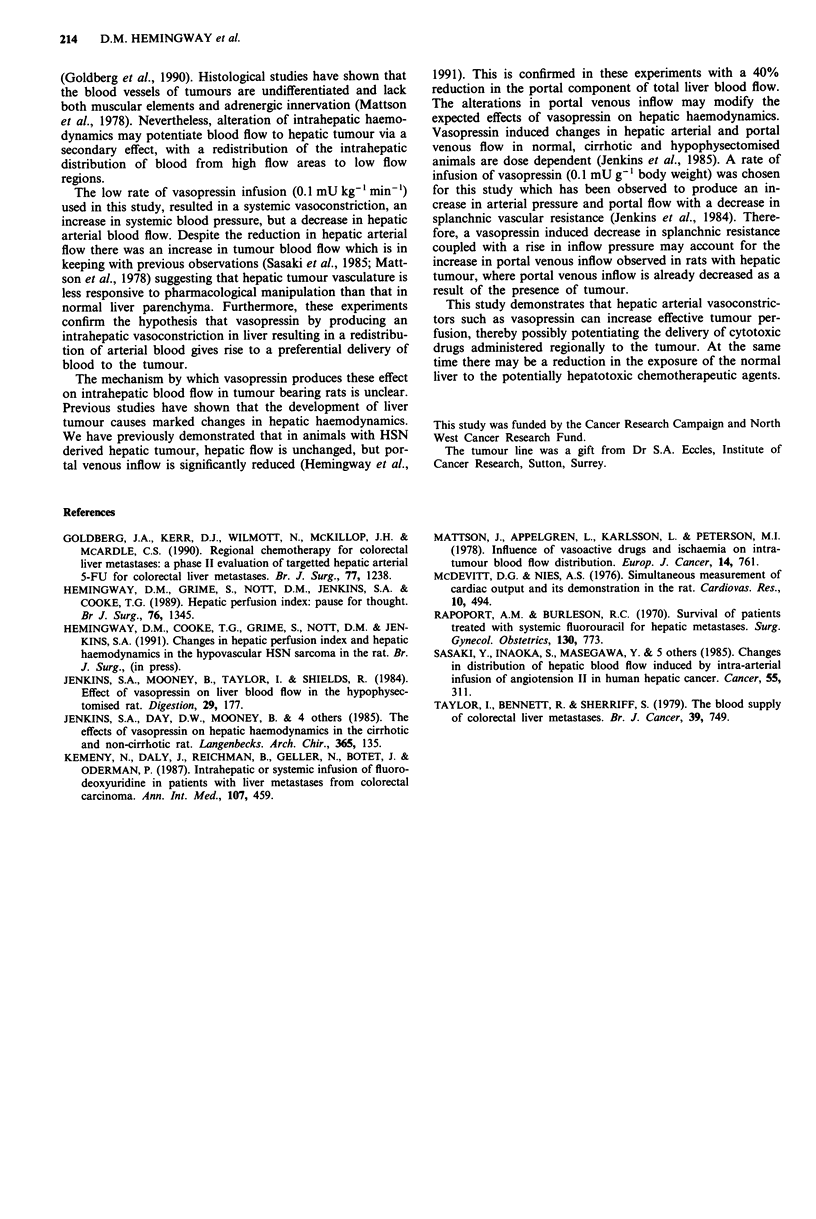# The effects of vasopressin infusion on hepatic haemodynamics in an experimental model of liver metastases.

**DOI:** 10.1038/bjc.1991.278

**Published:** 1991-08

**Authors:** D. M. Hemingway, D. Chang, T. G. Cooke, S. A. Jenkins

**Affiliations:** University Department of Surgery, Glasgow Royal Infirmary, UK.

## Abstract

Vasoactive drugs have a variety of effects upon splanchnic and hepatic haemodynamics which may alter tumour blood flow and potentiate the delivery of a chemotherapeutic drug to hepatic tumour. We have investigated the effects of vasopressin infusion on hepatic tumour blood flow in an experimental model of liver tumour. Hepatic tumour was induced by the intraportal inoculation of HSN sarcoma cells. Hepatic and splanchnic blood flow was determined using a dual reference microsphere technique before and after an intravenous infusion of vasopressin at a dose of 0.1 mU kg-1 min-1 for 10 min. There was a significant increase in systemic arterial blood pressure associated with a rise in portal venous inflow (P less than 0.01, Wilcoxen Signed rank Test) and a significant fall in hepatic arterial flow (P less than 0.05). The tumour: liver blood flow ratio was significantly increased by vasopressin infusion (P less than 0.02). Vasopressin infusion decreases hepatic arterial flow and increases tumour blood flow which may potentiate the delivery of a regionally delivered chemotherapeutic drug to hepatic tumour.


Br. J. Cancer (1991), 64, 212-214 © Macmillan Press Ltd., 1991

### The effects of vasopressin infusion on hepatic haemodynamics in an experimental model of liver metastases

D.M. Hemingway[1], D. Chang[2], T.G. Cooke[1] & S.A. Jenkins[1]

[1]*University Departments of Surgery, Glasgow Royal Infirmary and* [2]*Royal Liverpool Hospital, UK.*

**Summary** Vasoactive drugs have a variety of effects upon splanchnic and hepatic haemodynamics which may alter tumour blood flow and potentiate the delivery of a chemotherapeutic drug to hepatic tumour.

We have investigated the effects of vasopressin infusion on hepatic tumour blood flow in an experimental model of liver tumour.

Hepatic tumour was induced by the intraportal inoculation of HSN sarcoma cells. Hepatic and splanchnic blood flow was determined using a dual reference microsphere technique before and after an intravenous infusion of vasopressin at a dose of 0.1 mU $kg^{-1}min^{-1}$ for 10 min.

There was a significant increase in systemic arterial blood pressure associated with a rise in portal venous inflow ($P<0.01$, Wilcoxen Signed rank Test) and a significant fall in hepatic arterial flow ($P<0.05$). The tumour: liver blood flow ratio was significantly increased by vasopressin infusion ($P<0.02$). Vasopressin infusion decreases hepatic arterial flow and increases tumour blood flow which may potentiate the delivery of a regionally delivered chemotherapeutic drug to hepatic tumour.

Up to 60% of patients with colorectal cancer develop liver metastases which are rarely resectable surgically (Rapoport & Burleson, 1970). Systemic or regional chemotherapy is often used to treat these patients, but is relatively ineffective particularly with respect to survival. The poor response to chemotherapy may be a reflection of the hypovascular nature of most liver metastases which prevents access of therapeutic drug to the tumour (Kemeny *et al.*, 1987; Taylor *et al.*, 1979).

Vasoactive drugs have a variety of effects on splanchnic and hepatic haemodynamics which may alter tumour blood flow and potentiate the delivery of a chemotherapeutic drug to liver tumour (Mattson *et al.*, 1978). Vasopressin, a potent vasoconstrictor, reduces portal pressure in portal hypertensive patients and is used clinically to control variceal haemorrhage. However, the effects of vasopressin on hepatic haemodynamics varies with its rate of infusion. At low rates of infusion vasopressin has been demonstrated to decrease portal venous flow but increase hepatic arterial flow, whilst at higher rates of infusion these effects are reversed (Jenkins *et al.*, 1984; 1985).

We have developed a model of liver metastases in the rat using the HSN sarcoma. Our previous studies have demonstrated that tumour development in this model is associated with a decrease in portal venous inflow with no change in hepatic arterial flow. The tumours are supplied entirely from the hepatic artery, are histologically hypovascular compared to the surrounding liver parenchyma and do not show arteriosystemic shunting (Hemingway *et al.*, 1989). The model therefore displays many of the characteristics of human colorectal liver metastases and may prove useful in evaluating whether vasoactive drugs can potentiate delivery of cytotoxic drug to liver tumour. In this study we have investigated the effects of a systemic infusion of vasopressin on hepatic and splanchnic haemodynamics in rats with overt liver tumour.

### Methods

### *Tumour induction*

HSN sarcoma cells were grown in Dulbecco's modified Eagles Medium (Sigma, UK) supplemented with 10% foetal calf serum at 37°C in a humidified atmosphere of 5% $CO_2$ in an incubator. Metastases were induced by the intraportal inoculation of $10^6$ HSN sarcoma cells, trypsinised from a confluent monolayer, in Lister rats. Our previous studies have shown that discrete liver metastases are constantly present 3 weeks after the inoculation of these tumour cells.

### *Hepatic haemodynamics*

Organ blood flow, before and after the infusion of vasopressin, were measured using a dual microsphere reference method.

### *Effects of vasopressin*

One hundred thousand 57 Co microspheres (Nentrac, Dupont, Germany) suspended in normal saline with 0.01% Tween were injected over 20 s via a cannula (Portex, Hythe, UK, outside diameter 0.61 mm) screened into position in the left ventricle using a Siemens Image Intensifier (Siemens, Germany). A reference sample of blood was withdrawn from the right femoral artery starting 10 s before the injection of microspheres and continuing for 40 s after the injection. The withdrawal rate was constant at 1 ml per min. Arterial blood pressure was monitored using a strain gauge transducer and Gould pen recorder (Gould Medical, Lutterworth, UK) attached to a cannula in the left femoral artery.

In ten rats vasopressin was infused via the right femoral vein at a dose of 0.1 mU $kg^{-1}$ $min^{-1}$, for 10 min at a constant rate of 0.2 ml per min. Control rats received similar infusion of the same volume of isotonic saline ($n = 5$). At the end of the infusion of vasopressin or saline a second injection of 51 Cr microspheres, was given via the ventricular cannula and a second reference sample obtained as described previously. Five minutes later the animals were killed, the liver and splanchnic organs removed, weighed and the radioactivity counted on a gamma well counter (Packard, UK) together with the reference samples. The counts were corrected for spillover between the two channels. Results were rejected if renal blood flow between the right and left kidneys differed by greater than 10% or if any sample contained less than 400 particles, since this indicates inadequate ventricular mixing of macrospheres.

Organ blood flow was calculated using the method of McDevitt and Nies (1976):

$$\text{Organ blood flow} = \frac{\text{organ counts}}{\text{reference counts}} \times \text{reference withdrawal rate}$$

Hepatic arterial flow was calculated from the counts in the liver. Portal venous inflow was calculated from counts in the

Correspondence: T.G. Cooke, University Department of Surgery, Royal Infirmary, Glasgow G31 2ER, UK.
Received 19 December 1990; and in revised form 2 April 1991.

splanchnic organs draining into the portal vein. Hepatosplanchnic flow is the sum of hepatic arterial and portal venous inflows. Hepatic tumour was dissected from the surrounding normal liver, both weighed and counted separately, and the results expressed as a ratio of tumour:liver blood flow in ml min$^{-1}$ g$^{-1}$ tissue.

Statistical differences before and after infusion of vasopressin or saline were evaluated using the non-parametric Wilcoxen signed rank test.

### Results

The mean hepatic replacement by tumour was 27.12%. The haemodynamic effects of vasopressin infusion were as follows.

### *Changes in blood flow*

Hepatic arterial flow (Figure 1) was significantly decreased from 3.28 (1.82) ml min$^{-1}$ (Median (interquartile range)) to 1.65 (2.37) ml min$^{-1}$ after vasopressin infusion ($P<0.05$). However, portal venous inflow (Figure 2) increased significantly from 2.12 (1.93) to 5.38 (7.08) ml min$^{-1}$ ($P<0.01$). The infusion of vasopressin had no significant effect on hepatosplanchnic flow 5.54 (2.44) ml min$^{-1}$ to 7.52 (5.01) ml min$^{-1}$.

### *Tumour: liver flow*

The tumour:liver blood flow ratio rose significantly from a median of 0.38: one before, to 1.49: one following the infusion of vasopressin. This represents an effective doubling of tumour blood flow ($P=0.02$) (Figure 3).

### *Arterial blood pressure*

Arterial blood pressure rose from 95.5 (29.5) mmHg to 113 mmHg after vasopressin infusion ($P<0.01$).

### *Effects of saline infusion*

Saline infusion had no significant effect on hepatic arterial, portal venous or hepatosplanchnic flow. The tumour:liver blood flow ratio and systemic blood pressure were also unchanged (Table I).

**Figure 1** Change in hepatic arterial flow with vasopressin infusion ($P<0.02$ Wilcoxen signed rank test). Horizontal bar = median.

**Figure 2** Change in portal venous inflow with vasopressin infusion ($P<0.01$ Wilcoxen signed rank test). Horizontal bar = median.

### Discussion

The efficacy of regional chemotherapy may be improved by pharmacological manipulation of hepatic blood flow with vasoactive agents which by altering intrahepatic haemodynamics may potentiate the blood flow to liver tumour

**Figure 3** Change in the tumour:liver blood flow ratio after vasopressin infusion ($P=0.02$ – Wilcoxen signed rank test). Horizontal bar = median.

**Table I** Change in hepatic arterial flow, portal venous inflow, hepatosplanchnic flow, tumour:liver blood flow ratio and systemic blood pressure after saline infusion (0.2 ml min$^{-1}$).

| | *Saline (0.2 ml min$^{-1}$)* | |
|---|---|---|
| | *Pre-infusion* | *Post-infusion* |
| Hepatic arterial flow (ml min$^{-1}$) | 1.70 ( 0.59) | 1.41 (0.36) |
| Venous inflow (ml min$^{-1}$) | 5.31 ( 5.2) | 6.53 (3.15) |
| Hepatosplanchnic flow (ml min$^{-1}$) | 7.84 ( 4.42) | 7.89 (3.58) |
| Tumour:liver ratio | 0.58:1 | 0.60:1 |
| Systemic blood pressure (mmHg) | 97.3 (26.7) | 100.4 (39.6) |

(Figures are median (interquartile range))

(Goldberg *et al.*, 1990). Histological studies have shown that the blood vessels of tumours are undifferentiated and lack both muscular elements and adrenergic innervation (Mattson *et al.*, 1978). Nevertheless, alteration of intrahepatic haemodynamics may potentiate blood flow to hepatic tumour via a secondary effect, with a redistribution of the intrahepatic distribution of blood from high flow areas to low flow regions.

The low rate of vasopressin infusion ($0.1\ \mathrm{mU\ kg^{-1}\ min^{-1}}$) used in this study, resulted in a systemic vasoconstriction, an increase in systemic blood pressure, but a decrease in hepatic arterial blood flow. Despite the reduction in hepatic arterial flow there was an increase in tumour blood flow which is in keeping with previous observations (Sasaki *et al.*, 1985; Mattson *et al.*, 1978) suggesting that hepatic tumour vasculature is less responsive to pharmacological manipulation than that in normal liver parenchyma. Furthermore, these experiments confirm the hypothesis that vasopressin by producing an intrahepatic vasoconstriction in liver resulting in a redistribution of arterial blood gives rise to a preferential delivery of blood to the tumour.

The mechanism by which vasopressin produces these effect on intrahepatic blood flow in tumour bearing rats is unclear. Previous studies have shown that the development of liver tumour causes marked changes in hepatic haemodynamics. We have previously demonstrated that in animals with HSN derived hepatic tumour, hepatic flow is unchanged, but portal venous inflow is significantly reduced (Hemingway *et al.*, 1991). This is confirmed in these experiments with a 40% reduction in the portal component of total liver blood flow. The alterations in portal venous inflow may modify the expected effects of vasopressin on hepatic haemodynamics. Vasopressin induced changes in hepatic arterial and portal venous flow in normal, cirrhotic and hypophysectomised animals are dose dependent (Jenkins *et al.*, 1985). A rate of infusion of vasopressin ($0.1\ \mathrm{mU\ g^{-1}}$ body weight) was chosen for this study which has been observed to produce an increase in arterial pressure and portal flow with a decrease in splanchnic vascular resistance (Jenkins *et al.*, 1984). Therefore, a vasopressin induced decrease in splanchnic resistance coupled with a rise in inflow pressure may account for the increase in portal venous inflow observed in rats with hepatic tumour, where portal venous inflow is already decreased as a result of the presence of tumour.

This study demonstrates that hepatic arterial vasoconstrictors such as vasopressin can increase effective tumour perfusion, thereby possibly potentiating the delivery of cytotoxic drugs administered regionally to the tumour. At the same time there may be a reduction in the exposure of the normal liver to the potentially hepatotoxic chemotherapeutic agents.

This study was funded by the Cancer Research Campaign and North West Cancer Research Fund.

The tumour line was a gift from Dr S.A. Eccles, Institute of Cancer Research, Sutton, Surrey.

### References

GOLDBERG, J.A., KERR, D.J., WILMOTT, N., MCKILLOP, J.H. & MCARDLE, C.S. (1990). Regional chemotherapy for colorectal liver metastases: a phase II evaluation of targetted hepatic arterial 5-FU for colorectal liver metastases. *Br. J. Surg.*, **77**, 1238.

HEMINGWAY, D.M., GRIME, S., NOTT, D.M., JENKINS, S.A. & COOKE, T.G. (1989). Hepatic perfusion index: pause for thought. *Br J. Surg.*, **76**, 1345.

HEMINGWAY, D.M., COOKE, T.G., GRIME, S., NOTT, D.M. & JENKINS, S.A. (1991). Changes in hepatic perfusion index and hepatic haemodynamics in the hypovascular HSN sarcoma in the rat. *Br. J. Surg.*, (in press).

JENKINS, S.A., MOONEY, B., TAYLOR, I. & SHIELDS, R. (1984). Effect of vasopressin on liver blood flow in the hypophysectomised rat. *Digestion*, **29**, 177.

JENKINS, S.A., DAY, D.W., MOONEY, B. & 4 others (1985). The effects of vasopressin on hepatic haemodynamics in the cirrhotic and non-cirrhotic rat. *Langenbecks. Arch. Chir.*, **365**, 135.

KEMENY, N., DALY, J., REICHMAN, B., GELLER, N., BOTET, J. & ODERMAN, P. (1987). Intrahepatic or systemic infusion of fluorodeoxyuridine in patients with liver metastases from colorectal carcinoma. *Ann. Int. Med.*, **107**, 459.

MATTSON, J., APPELGREN, L., KARLSSON, L. & PETERSON, M.I. (1978). Influence of vasoactive drugs and ischaemia on intratumour blood flow distribution. *Europ. J. Cancer*, **14**, 761.

MCDEVITT, D.G. & NIES, A.S. (1976). Simultaneous measurement of cardiac output and its demonstration in the rat. *Cardiovas. Res.*, **10**, 494.

RAPOPORT, A.M. & BURLESON, R.C. (1970). Survival of patients treated with systemic fluorouracil for hepatic metastases. *Surg. Gynecol. Obstetrics*, **130**, 773.

SASAKI, Y., INAOKA, S., MASEGAWA, Y. & 5 others (1985). Changes in distribution of hepatic blood flow induced by intra-arterial infusion of angiotension II in human hepatic cancer. *Cancer*, **55**, 311.

TAYLOR, I., BENNETT, R. & SHERRIFF, S. (1979). The blood supply of colorectal liver metastases. *Br. J. Cancer*, **39**, 749.